# A Hierarchical Visual Navigation Algorithm for UAVs Integrating Artificial Potential Field and Deep Reinforcement Learning

**DOI:** 10.3390/s26165196

**Published:** 2026-08-17

**Authors:** Dongliang Wang, Yongqiang Jin, Weicheng Luo, Yijing Yang, Senyi Zhang, Yong Gao

**Affiliations:** 1 College of Engineering, Shantou University, Shantou 515063, China; dlwang@stu.edu.cn (D.W.); 25yqjin@stu.edu.cn (Y.J.); 19wcluo@stu.edu.cn (W.L.); 24yjang@stu.edu.cn (Y.Y.); 24syzhang@stu.edu.cn (S.Z.); 2 Guangdong Institute of Modern Agricultural Equipment, Guangzhou 510630, China

**Keywords:** UAV navigation, deep reinforcement learning, visual guidance, artificial potential field

## Abstract

To address the challenge of rapid and precise obstacle avoidance for unmanned aerial vehicles (UAVs) in complex urban environments, rugged canyons, and other unstructured environments, this paper proposes a vision-based navigation algorithm. By combining the strengths of deep reinforcement learning (DRL) and convolutional neural networks (CNNs), this algorithm enables efficient navigation and obstacle avoidance in dynamic environments. First, to improve training efficiency, an autoencoder is used to extract latent spatial vectors from depth images, which are then used as input features for DRL. Second, an artificial potential field (APF) is introduced into the reward function to enhance obstacle avoidance performance in dynamic environments. Third, a CNN-based adaptive mode-switching mechanism is designed to meet navigation requirements under different environmental conditions. This mechanism can automatically identify environmental features based on real-time input data and dynamically adjust the UAV’s navigation strategy. To evaluate the proposed method, simulation experiments were conducted in static and dynamic scenarios, together with a preliminary indoor flight test. Under the evaluated conditions, the proposed method achieved favorable navigation success rates and path efficiency compared with the selected visual DRL baselines. The results also indicate cross-scenario transferability to the tested environments without environment-specific retraining.

## 1. Introduction

Over the past decade, drones have made significant contributions through their navigation technology in agriculture [1], surveillance [2], logistics [3], search and rescue [4], and emergency response [5], as well as military applications [6]. In practical applications, UAVs often need to perform navigation tasks in complex environments characterized by dense, dynamic, and unpredictable obstacles [7]. Such unknown and unstructured environments place higher demands on the perception and decision-making capabilities of UAVs, particularly in terms of real-time, accurate obstacle detection and navigation [8]. Therefore, autonomous navigation capabilities are crucial for the practical application of UAVs [9].

Extensive research has been conducted on autonomous UAV navigation in three-dimensional dynamic environments, demonstrating that model-based or rule-based methods exhibit high reliability and effectiveness [10]. These methods typically rely on navigation maps for path planning, encompassing both global and local mapping techniques. For example, an omnidirectional vision system can be used to construct a global environmental map, and corresponding velocity commands can be generated using a genetic control network model [11]. First, an initial trajectory is planned without considering obstacles; then, obstacles are detected in real time during flight and the map is updated; finally, the original trajectory is dynamically adjusted using a Euclidean signed distance field [12]. The Artificial Potential Field (APF) method is used to model the target as an attractive field and obstacles as repulsive fields, thereby enabling safe obstacle avoidance and precise navigation [13]. Furthermore, path planning can be defined as a nonlinear optimization problem, incorporating an obstacle gradient field into the optimization model to guide the UAV in generating trajectories that balance safety and smoothness [14]. However, such methods typically rely on sensors to obtain precise environmental information. In practical applications, due to limitations in perception capabilities, it is often difficult to accurately estimate the positions of obstacles [15], thereby affecting the system’s adaptability in unknown or dynamic environments.

Deep Reinforcement Learning (DRL) enables end-to-end autonomous navigation of drones by directly mapping sensor data to control commands and optimizing them using a reward function, without the need for explicit environmental mapping or precise obstacle localization [16,17]. For example, residual networks can be used to extract features from RGB images and fuse them with other sensor data to serve as state inputs for DRL [18]. Furthermore, based on the actor–critic framework, RGB images can be directly fed into the actor network to generate actions [19]. To address the lack of depth information in RGB images, some studies have introduced depth estimation techniques. For example, Generative Adversarial Networks (GANs) can be used to estimate depth information from RGB images for training Deep Q-Networks (DQNs) [20]. Another approach directly utilizes depth images for navigation. Previous research has developed a navigation method that integrates a convolutional neural network (CNN) into the Soft Actor–Critic (SAC) framework to process depth images [21]. Similarly, autoencoders can be used to extract latent representations from sequences of depth images, thereby enhancing spatiotemporal memory capabilities [22]. In addition to these architectural improvements, causal feature selection (CFS) has been introduced to enhance sensitivity to critical obstacle information [23]. However, in unstructured environments, the generalization capabilities of existing methods remain insufficient: agents that perform well in training scenarios often experience a significant drop in performance when encountering unknown obstacles or environmental changes [24].

In deep reinforcement learning (DRL), the generalization problem primarily stems from its reliance on the “independent and identically distributed” (i.i.d.) assumption [25], which often fails due to differences between the training and testing environments [26]. For example, in the reward function designs presented in [22,23], the agent receives a positive reward when moving toward a target and a penalty when approaching an obstacle [27]. Such rewards are typically calculated based solely on the closest obstacle point in the current depth image, resulting in obstacles with different geometric shapes potentially generating identical reward signals. In reality, however, drones need to execute different obstacle-avoidance strategies based on the specific shape of the obstacle. In contrast, the APF method, as a classic potential field control method, is suitable for both global path planning and local real-time navigation [28]. Its algorithmic structure possesses inherent generalization and scene transfer capabilities, which is why it has attracted widespread attention and been extensively applied in practice [29]. To improve the generalization performance of Deep Reinforcement Learning (DRL), related studies [30,31] have incorporated APF (Artificial Potential Field) modeling into the design of DRL reward functions, effectively enhancing obstacle avoidance capabilities in unknown environments. However, in dense and cluttered environments, the velocity commands output by DRL models often couple navigation objectives with obstacle avoidance requirements, leading to oscillatory behavior in the generated commands [32]. This manifests as the drone repeatedly approaching and then retreating from obstacles, resulting in unstable motion patterns.

The core issue behind oscillatory DRL velocity commands lies in the inherent trade-off between navigating toward the goal and avoiding obstacles during velocity decision-making [33]. Since the DRL model is simultaneously responsible for generating and coupling both navigation and obstacle avoidance velocities, its output prioritizes moving away from detected obstacles to ensure safety [34]. To address this problem, a promising approach involves adopting a hierarchical decision-making structure, where an independent module handles goal-oriented navigation, allowing the DRL model to focus specifically on obstacle avoidance [35]. For instance, in the domain of robotic arm control, a dual-mode hybrid control architecture has been developed that integrates a global motion planner, which does not consider obstacles, with a local obstacle avoidance strategy based on DRL, and achieves automatic switching between the two modes by setting a distance threshold [36]. In the field of automated guided vehicles, a DRL-based control switching mechanism has been designed that is capable of dynamically selecting either the Timed Elastic Band global planner or a DRL local planner based on sensor information [37]. In a simulation environment, a differential goal-driven mechanism has been introduced to guide the robot toward the target point while utilizing a DRL model to avoid dynamic obstacles, and a hidden Markov model has been employed to achieve the fusion of multi-source decisions [38]. These methods use decision decoupling and hierarchical control mechanisms to enable DRL training using only obstacle-avoidance-related data, thereby mitigating gradient conflicts and improving policy smoothness [39]. However, existing studies have generally addressed reward design or hierarchical decision-making separately, while their joint use for improving cross-scenario navigation and reducing velocity oscillations remains insufficiently explored.

Building on the aforementioned research, this paper proposes a hierarchical navigation framework that utilizes convolutional neural networks (CNNs) and deep reinforcement learning (DRL) for UAV control, combined with the APF (Artificial Potential Field) algorithm to optimize the control algorithm, in order to address the navigation challenges UAVs face in unknown, unstructured environments. This method utilizes visual and inertial sensors for navigation, without relying on external positioning systems or pre-constructed maps. The framework uses an autoencoder to extract features from collected depth images for DRL training. The framework also includes a CNN acting as a switching module, which takes the current depth image as input and outputs an obstacle-avoidance signal. When no obstacle-avoidance signal is detected, the UAV moves directly toward the target using a feedforward control mechanism; otherwise, it executes the control commands generated by the DRL policy.

The main contributions of this paper are summarized as follows:

- This paper proposes a map-free, depth-vision-based hierarchical navigation framework for unmanned aerial vehicles (UAVs). The framework generates navigation commands directly from the UAV’s depth observations, target direction, and current velocity, without relying on explicit environmental mapping, pre-built maps, or external positioning systems. A CNN-based switching module dynamically activates a local depth reinforcement learning obstacle avoidance strategy based on current visual observations, thereby enabling fully onboard autonomous navigation.
- We designed a non-sparse reward function based on the Artificial Potential Field (APF) that integrates information from multiple obstacle points within the safety margin. In the evaluated scenarios, the configuration using this reward function achieved a numerically higher navigation success rate and better cross-scenario transfer performance than the corresponding configuration without it.
- We introduced a decision-decoupling mechanism through hierarchical control. A convolutional neural network (CNN)-based switching module separates goal-oriented control from local obstacle avoidance performed by the Soft Actor–Critic (SAC) policy. In the evaluated scenarios, the full APF+CNN configuration obtained lower JRMS values than the configurations without the complete hierarchical mechanism, suggesting reduced velocity oscillations under the tested conditions.

## 2. Materials and Methods

### 2.1. Problem and Approach

In unstructured environments, drones must navigate toward high-precision targets while simultaneously avoiding dynamic obstacles, which poses significant challenges for perception and decision-making systems. Due to environmental dynamics and computational complexity, traditional methods often result in inefficient navigation or compromised safety. To address this issue, this study proposes a hierarchical navigation framework for drones, whose core concept is to organically integrate goal-oriented global navigation with local obstacle avoidance strategies through a dynamic switching mechanism.

**Figure 1:**
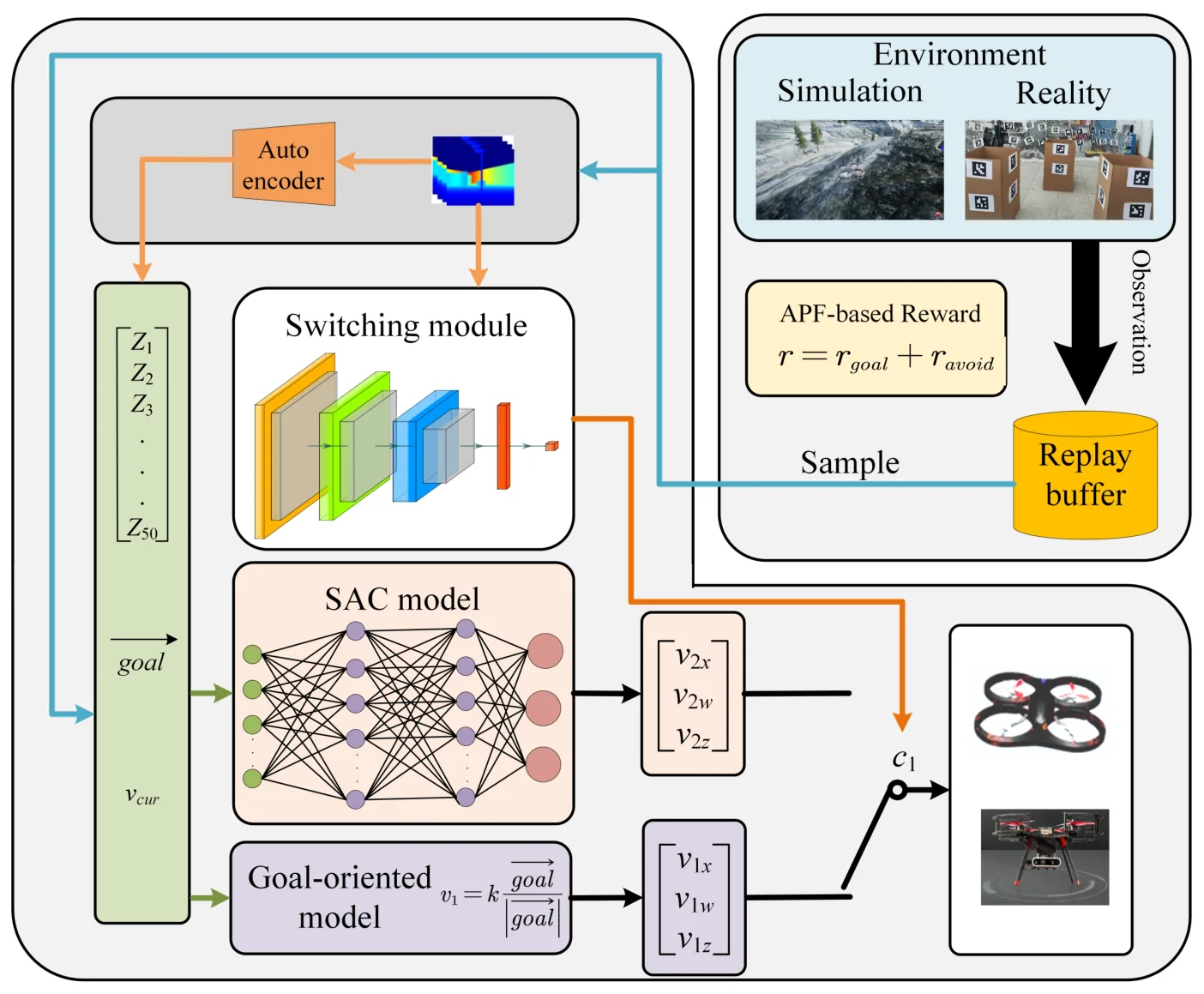
A hierarchical navigation framework for UAVs.

As shown in Figure 1, this framework consists of two core modules: perception and decision-making. The obstacle avoidance module on the left uses the SAC model combined with an autoencoder (AE) to generate local velocity commands for obstacle avoidance. The goal-oriented module generates velocity commands toward the target based on the target direction. The environment interaction module on the right is responsible for data acquisition and reward calculation, and dynamically selects the final velocity commands through a CNN-based gated switching layer (the switching module shown in the figure), thereby enabling safe and efficient autonomous navigation.

In the obstacle-avoidance module, the system first uses an autoencoder with an encoder–decoder architecture to extract a 50-dimensional latent feature vector ($Z_{1}$ to $Z_{50}$) from the depth images. This vector, combined with the goal direction ($\vec{\mathrm{goal}}$) and the current velocity ($v_{\mathrm{cur}}$), forms the state input. This input is then processed by the SAC policy network to output a three-dimensional obstacle avoidance velocity command. To enhance training efficiency, the reward function adopts a structured reward design based on APF, decomposing the navigation task into goal-approaching reward and obstacle-avoidance reward, which jointly constitute a non-sparse reward signal.

The gated switching module takes the current depth image as input and generates a control signal through a lightweight CNN. The final velocity output is selected by the gated switch controlled by the control signal $c_{1}$ between the direct goal-oriented command or the obstacle avoidance command. When the CNN determines that no obstacles are present in the image, the system prioritizes the goal-oriented command; when an obstacle is detected, the obstacle avoidance command is activated. All interaction data is stored in the experience replay buffer for continuous optimization of network performance.

### 2.2. Assumptions

The implementation of the algorithm proposed in this paper is based on the following basic assumptions:

1. The drone can rely entirely on its onboard depth camera and speed sensors for environmental perception. Obstacle detection is achieved through stereo vision depth sensing.
2. The depth camera on the drone faces forward; by combining yaw rotation with forward motion, the drone can move backward.
3. Suppose that the velocity of a drone operating in a three-dimensional velocity space is subject to the following constraints: the forward velocity $v_{x}$ is restricted to the interval [0.0,1.5]m/s, the horizontal angular velocity $v_{\omega }$ is limited to the range [−π,π]rad/s, and the vertical velocity $v_{z}$ is restricted to the interval [−1.0,1.0]m/s.

### 2.3. Evaluation Metrics

In comparing the performance of different methods, we use the following quantitative evaluation metrics:

1. Success Rate: This metric represents the proportion of experiments in which a drone successfully navigated from a random starting point to a target location without collisions within a specified time period [40]. This metric reflects the reliability of the navigation algorithm.
2. Path Length-Weighted Success Rate (SPL): This metric combines success rate and path efficiency [40], and is calculated as follows:
  (1) $$\mathrm{SPL}=\frac{1}{N}\sum_{i=1}^{N}S_{i}\frac{l_{\mathrm{opt}}}{\max\left(l_{\mathrm{opt}},l_{\mathrm{nav}}\right)},$$
  where $S_{i}$ indicates whether the *i*-th experiment was successful (1 for success, 0 for failure), $l_{\mathrm{opt}}$ is the shortest straight-line distance from the starting point to the target, $l_{\mathrm{nav}}$ is the actual navigation path length of the UAV, and *N* is the total number of experiments.
3. Extra Distance: This metric quantifies the ratio of the actual length of the UAV’s flight path to the straight-line distance between the starting point and the target location [41]. It reflects the degree of optimization of the navigation path; a lower value indicates that the path is closer to the optimal one.
4. Average Speed: This metric is used to measure the average speed of the drone during the experiment. It serves as a measure of the navigation algorithm’s efficiency and dynamic performance [41].
5. Root Mean Square Jerk (JRMS): This metric is used to quantify the smoothness of a drone’s motion [42], and is defined as follows:
  (2) $$\mathrm{JRMS}=\sqrt{\frac{1}{N}\sum_{i=1}^{N}j_{i}^{2}},$$
  Here, *N* denotes the number of time steps in the navigation task, $j_{i}=\frac{a_{i+1}-a_{i}}{\Delta t}$, $j_{i}$ represents the rate of change of acceleration, and $a_{i}$ represents the acceleration at the *i*th time step.
6. Average Minimum Obstacle Distance (AMOD): Obstacle avoidance metrics are commonly used to evaluate navigation safety [43,44]. AMOD measures the average distance between a drone and relevant dynamic obstacles at the point of closest approach during a successful flight:
  (3) $$\mathrm{AMOD}=\frac{1}{\left|V\right|}\sum_{j\in V}\min_{i\in S,\;t}d_{i,j,t},$$
  Here, S is the set of successful flight segments, V is the set of relevant dynamic obstacles, and $d_{i,j,t}$ represents the distance between the UAV and an obstacle. A larger AMOD value indicates a higher average safety margin.
7. Proximity Violation Rate (PVR): Based on the proximity violation metric for dynamic collaborative navigation [45], PVR measures the proportion of time steps during the recorded period in which the distance between the UAV and the nearest dynamic obstacle is less than a preset proximity threshold.
  (4) $$\mathrm{PVR}\left(r_{\mathrm{prox}}\right)=\frac{1}{\left|S\right|}\sum_{i\in S}\frac{1}{T_{i}}\sum_{t=1}^{T_{i}}I\;\left(d_{i,t}^{\mathrm{near}}\leq r_{\mathrm{prox}}\right)\times 100\%,$$
  Here, $T_{i}$ denotes the number of time steps in the record, $d_{i,t}^{\mathrm{near}}$ is the distance to the nearest obstacle, $r_{\mathrm{prox}}$ is the proximity threshold, and I(·) is the indicator function. The lower the PVR, the shorter the duration of continuous close-range exposure.

## 3. Hierarchical Framework Design

This section systematically describes the hierarchical framework for autonomous UAV navigation proposed in this paper. Our framework deeply integrates a CNN-based switching mechanism with a deep reinforcement learning (DRL) module specifically designed for obstacle avoidance, aiming to comprehensively enhance navigation efficiency and safety. First, we detail the network architecture of the switching module and its specific training strategy. We then focus on modeling the navigation task as a partially observable Markov decision process (POMDP) and implementing it using the SAC algorithm. This includes the design and integration of an APF-based reward function, as well as the specific methodology for the collaborative training of the SAC algorithm and the switching module.

### 3.1. Switching Module

During drone navigation, if no obstacles are detected in the central region of the depth image, the drone should be controlled to proceed directly toward the target to avoid velocity oscillations caused by a conflict between approaching the target and avoiding obstacles. Therefore, a dedicated module is needed to determine the relative distance between the central image region and potential obstacles. However, in complex environments with severe image distortion, methods based solely on depth image pixel values exhibit low robustness [46]. To address this issue, this study introduces a convolutional neural network (CNN) as a switching decision module. By integrating this CNN-based switching module, the UAV proceeds directly toward the target when no obstacles are detected in the central region of the depth image; otherwise, it executes the obstacle-avoidance velocity commands generated by the deep reinforcement learning (DRL) model. This decision-making strategy can be described as follows:

(5) $$v=\left[\begin{array}{c} v_{x}\\ v_{\omega }\\ v_{z} \end{array}\right]=c_{1}\left[\begin{array}{c} v_{1x}\\ v_{1\omega }\\ v_{1z} \end{array}\right]+\left(1-c_{1}\right)\left[\begin{array}{c} v_{2x}\\ v_{2\omega }\\ v_{2z} \end{array}\right],$$

In particular, $v={\left[v_{x},v_{\omega },v_{z}\right]}^{T}$ represents the final velocity command output sent to the UAV, where $v_{x}$ denotes the forward velocity, $v_{\omega }$ denotes the yaw angular velocity, and $v_{z}$ denotes the vertical velocity. The binary control variable $c_{1}\in \left\{0,1\right\}$ is output by the switching module: when $c_{1}=1$, it indicates that obstacle avoidance is not required, and the system adopts the target-oriented velocity vector $v_{1}={\left[v_{1x},v_{1\omega },v_{1z}\right]}^{T}$ to guide the UAV toward the target; when $c_{1}=0$, it indicates that obstacle avoidance is required, and the system activates the obstacle-avoidance velocity vector $v_{2}={\left[v_{2x},v_{2\omega },v_{2z}\right]}^{T}$ generated by the SAC module.

For this specific task, the CNN employs a binary classification architecture. The network’s loss function uses binary cross-entropy (BCE), defined as follows:

(6) $$\mathrm{BCE}=-\frac{1}{N}\sum_{i=1}^{N}\left[y_{i}\log\left(p_{i}\right)+\left(1-y_{i}\right)\log\left(1-p_{i}\right)\right],$$

where *N* is the total number of training samples, and $y_{i}\in \left\{0,1\right\}$ and $p_{i}$ are the true label and predicted probability for the *i*th sample, respectively.

The CNN architecture consists of one convolutional layer, one pooling layer, and two fully connected layers. The convolutional layer extracts features from the input depth image, and the pooling layer subsequently reduces the dimensionality of these features. Next, the two fully connected layers further process the feature information, and the final output generates classification probabilities via the sigmoid function.

We collected diverse scene data by having a drone move autonomously and randomly within a simulated environment. The simulated environment provides precise point-cloud information about obstacle surfaces, allowing the exact distance between the drone and the obstacles to be calculated while depth images are acquired. If the distance between the drone and any point on an obstacle is less than a preset safety threshold, the depth image is labeled as “close to obstacle” (denoted as $c_{1}=0$); otherwise, it is labeled as “far from obstacle” (denoted as $c_{1}=1$). This binary classification approach provides a clear objective for training the CNN. The hyperparameters used to train the CNN are listed in Table 1.

### 3.2. Deep Reinforcement Learning

#### 3.2.1. Soft Actor–Critic

In this study, the UAV relies only on its onboard sensors to accomplish obstacle avoidance and navigation tasks. This constraint means that the UAV can only perceive a limited portion of its environment, precluding access to comprehensive environmental data. Thus, the obstacle avoidance navigation problem is modeled as a POMDP. Formally, the POMDP is defined by a six-tuple $\left(S,\;A,\;T,\;R,\;\Omega ,\;O\right)$, where *S* represents the state space, *A* corresponds to the action space, *T* describes the state transition probability function, *R* is the reward function that maps state-action pairs to rewards, Ω denotes the observation space, and *O* represents the observation distribution given a state, with observation following this distribution $\left(o∼O\left(s\right)\right)$.

In terms of implementation, the adopted SAC+AE architecture [47] takes four consecutive depth images, the target direction, and the UAV’s current velocity as inputs, and outputs a three-dimensional velocity command. As shown in Figure 1, the model structure primarily consists of a perception processing module (AE) and a decision-making module (SAC). The encoder comprises four convolutional layers, each followed by a ReLU activation function, and ultimately outputs a 50-dimensional latent representation through a fully connected layer. The actor network consists of the encoder and the policy network connected in series, while the critic network integrates the encoder with the Q-value network. The policy network is a multi-layer perceptron that takes as input the 50-dimensional latent representation, the target direction vector, and the current velocity, and outputs a three-dimensional mean vector and a covariance matrix. These parameters are used together to fit a Gaussian action distribution, thereby generating control commands. In addition to receiving the same inputs as the policy network, the Q-value network also incorporates the actions output by the policy network and ultimately outputs the corresponding one-dimensional Q-values.

At each time step *t*, the observation space is defined as $o^{t}={\left[o_{z}^{t},o_{g}^{t},o_{v}^{t}\right]}^{T}$, where $o_{z}^{t}$ represents the depth image information constructed from four consecutive frames, $o_{g}^{t}$ represents the direction vector from the current position to the target position, and $o_{v}^{t}={\left[v_{x}^{t},v_{\omega }^{t},v_{z}^{t}\right]}^{T}$ represents the drone’s current velocity. The action space is designed to be continuous and bounded. At each time step *t*, the policy network generates an action $a^{t}={\left[v_{x}^{\mathrm{cmd}},v_{\omega }^{\mathrm{cmd}},v_{z}^{\mathrm{cmd}}\right]}^{T}$, corresponding to the forward velocity, yaw angular velocity, and vertical velocity commands, respectively. This design ensures smooth motion while avoiding control instability caused by sudden changes in velocity.

#### 3.2.2. Reward Function

Our goal is to enable the policy to learn navigation behavior similar to that produced by the artificial potential field method. Therefore, based on the attraction and repulsion formulas of the artificial potential field, we designed a non-sparse reward function. The reward function is expressed as follows:

(7) $$r^{t}=r_{\mathrm{goal}}^{t}+r_{\mathrm{avoid}}^{t},$$

where $r^{t}$ is the total reward at time step *t*, $r_{\mathrm{goal}}^{t}$ and $r_{\mathrm{avoid}}^{t}$ are the goal-approaching reward and obstacle-avoidance reward at time step *t*, respectively. This formulation incorporates the dual influences of gravitational pull toward the target and repulsive forces away from obstacles, thereby enabling the UAV to navigate optimally toward the destination while effectively evading potential collisions.

In the APF method, the robot’s motion is determined by the superposition of attractive and repulsive potential fields. The expression for the attractive potential field force is:

(8) $$F^{att}\left(p\right)=-k_{a}∥p-g∥,$$

where *p* and *g* represent the robot’s position and the target point’s position, respectively. The coefficient $k_{a}$ is a weight that determines the strength of the attractive force.

In accordance with the formula for attractiveness, we design the reward function $r_{\mathrm{goal}}^{t}$ as follows:

(9) $$r_{\mathrm{goal}}^{t}=\left\{\begin{array}{cc} r_{\mathrm{reach}}, & 0<d^{t}\leq 1\\ k_{a}\left(d^{t-1}-d^{t}\right), & \left(d^{t-1}-d^{t}\right)>0\\ 0, & \left(d^{t-1}-d^{t}\right)\leq 0 \end{array}\right.,$$

where $d^{t}$ and $d^{t-1}$ denote the distances from the UAV to the target at time steps *t* and t−1, and $r_{\mathrm{reach}}$ denotes the reward for successfully reaching the target. The reward value $r_{\mathrm{goal}}^{t}$ is designed to guide the UAV to learn how to efficiently reach the target position. When the UAV approaches within 1 m of the target point, the task is considered complete, and the UAV receives a fixed reward value $r_{\mathrm{reach}}$. Otherwise, at each time step, the reward is calculated based on the change in distance between the UAV and the target: the closer the UAV gets to the target, the greater the reward; conversely, if the distance to the target increases, no reward is given. Specifically, the reward value is proportional to the distance moved by the UAV in the direction of the target, weighted by an attraction coefficient. This design encourages the UAV to move efficiently toward the target while avoiding ineffective or counterproductive movements.

The repulsive potential field function can be formulated as follows:

(10) $$F_{o}^{rep}\left(p\right)=\left\{\begin{array}{cc} k_{r}\left(\frac{1}{r}-\frac{1}{\rho_{0}}\right)\frac{1}{r^{2}}, & r\leq \rho_{0}\\ 0, & r>\rho_{0} \end{array}\right.,$$

where *p* and *o* represent the robot’s position and the obstacle’s location, r=∥p−o∥ is the distance between them, $k_{r}$ is the repulsive force coefficient, and $\rho_{0}$ is the influence range of the repulsive field.

In accordance with the formula for repulsive force, we designed the reward value $r_{\mathrm{avoid}}^{t}$ to help the UAV learn obstacle avoidance capabilities. The reward function is formulated as follows:

(11) $$r_{\mathrm{avoid}}^{t}=\left\{\begin{array}{cc} r_{\mathrm{collision}}, & \mathrm{if}\;∥p-o∥<d_{\mathrm{collision}}\\ -∥\sum_{j=0}^{M}F_{o}^{\mathrm{rep}}\left(p\right)∥, & \mathrm{if}\;d_{\mathrm{collision}}\leq ∥p-o∥<R\\ 0, & \mathrm{if}\;∥p-o∥\geq R \end{array}\right.,$$

where *p* and *o* denote the UAV position and the obstacle position, respectively; $d_{\mathrm{collision}}$ is the collision-distance threshold, *R* is the prescribed safety distance, and *M* is the number of obstacle points within that distance. When the distance between the UAV and an obstacle is less than $d_{\mathrm{collision}}$, the UAV receives a collision penalty $r_{\mathrm{collision}}$. When the distance is within the safe range *R*, the sum of repulsive forces from all obstacle points is calculated and incorporated into the reward value. This design encourages the UAV to maintain a safe distance from obstacles during flight, thereby promoting collision avoidance.

#### 3.2.3. SAC Training Policy

Since the velocity generated by the DRL policy is used solely for obstacle-avoidance navigation, data collection during model training occurs only when the CNN switches the current velocity command to the velocity generated by the DRL model. In each round, both the drone’s initial position and target position are randomly generated in three-dimensional space; both positions are located on either side of an obstacle, and the distance between the initial and target positions is maintained within a fixed range. During training, the obstacle surfaces are modeled using point clouds, and the reward values are calculated based on the position coordinates of the obstacles relative to the drone. It should be noted that only obstacle points within a certain range of the depth camera are considered. To improve training stability, the encoder parameters are updated only in the Critic network, while the encoder portion in the Actor network remains frozen. The detailed steps of the training process are shown in Algorithm 1, and the hyperparameters used for training are listed in Table 2.

The overall training procedure, which combines SAC with APF and CNN, is summarized in Algorithm 1. The algorithm first initializes the Actor, Critic, Encoder, Decoder, and the experience replay buffer *B*, and preloads the pre-trained CNN model. At the start of each training epoch, the system observes the initial observation $o^{1}$ and the initial image $I^{1}$. At each time step *t*, if the termination condition has not yet been met, an action $a^{t}$ is selected via the policy network $\pi_{\theta }$ based on the current observation $o^{t}$, while the CNN model computes the switching signal $c_{1}^{t}$ using $I^{t}$ to determine the speed control mode. Subsequently, the system receives the reward $r^{t}$, the next observation $o^{t+1}$, the next image $I^{t+1}$, and the completion flag $d^{t}$ from the environment. If $c_{1}^{t}$ is zero at this point—indicating the need to avoid an obstacle—the current transition experience $\left(o^{t},a^{t},r^{t},o^{t+1},d^{t}\right)$ is stored in buffer *B*. In the internal update loop, the algorithm randomly samples a batch of experiences from the buffer and uses them to update the parameters of the actor, critic, encoder, and decoder networks, thereby achieving end-to-end policy optimization and feature learning.
**Algorithm 1** Hierarchical UAV navigation training algorithm**Input:** Pre-trained CNN model, max_episodes, max_timesteps, max_update_times**Output:** Trained Actor, Critic, Encoder, Decoder 1:Initialize Actor, Critic, Encoder, Decoder, and replay buffer *B* 2:Load the pre-trained CNN model 3:**for** each episode from 1 to max_episodes **do** 4:   Observe the initial observation $o^{1}$ and image $I^{1}$ 5:   **for** each time step *t* from 1 to max_timesteps **do** 6:      **if** the terminal state is not reached **then** 7:         Select action $a^{t}∼\pi_{\theta }\left(\cdot |o^{t}\right)$ 8:         Compute switching signal $c_{1}^{t}$ using the CNN model with $I^{t}$ 9:         Obtain reward $r^{t}$, observation $o^{t+1}$, image $I^{t+1}$, and done signal $d^{t}$10:         **if** $c_{1}^{t}=0$ **then**11:            Store $\left(o^{t},a^{t},r^{t},o^{t+1},d^{t}\right)$ in *B*12:         **end if**13:         **for** each update iteration from 1 to max_update_times **do**14:            Sample a batch of experiences $\left(o,a,r,o^{\prime },d\right)$ from *B*15:            Update the Actor, Critic, Encoder, and Decoder16:         **end for**17:      **end if**18:   **end for**19:**end for**

## 4. Experiments and Results

This section describes the simulation environment and three evaluation scenarios. Results are reported separately for the static Playground and Canyon scenarios and for the dynamic Urban scenario. The static evaluation compares the proposed method with SAC+RAE and SAC+CFS, whereas the dynamic evaluation additionally includes SAC-CUPRL and CURL-Depth. Finally, ablation experiments quantify the contributions of the APF-based reward function and the CNN switching module.

### 4.1. Simulation Setup

#### 4.1.1. Simulation Environment

During the implementation of the DRL algorithm and CNN training, we utilized the AirSim simulator [48], which is built upon the Unreal Engine. This simulator provides a highly realistic environment for algorithm training. Furthermore, it was also employed to generate the training dataset for the CNN. Our neural networks were developed using a deep learning framework within a Linux environment and were trained on a high-performance PC equipped with an Intel i9-14900K processor and an NVIDIA Quadro RTX 4090 GPU.

#### 4.1.2. Scenarios

**Figure 2:**
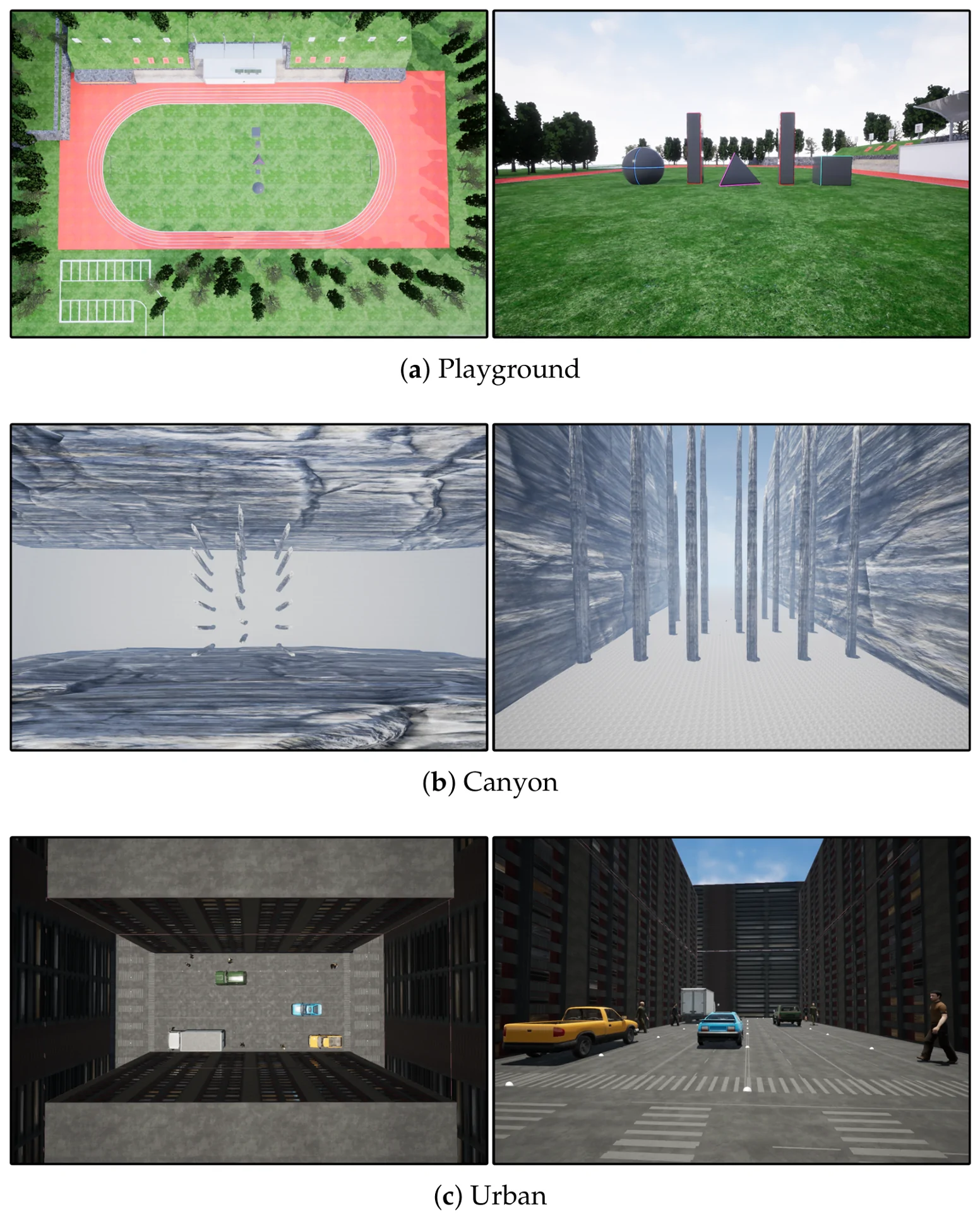
Three simulation scenarios: (**a**) playground; (**b**) canyon; (**c**) urban.

As shown in Figure 2, the experiments were conducted in three scenarios to evaluate the UAV navigation algorithm under different obstacle configurations and motion conditions:

1. Playground Scene: This scene features an open soccer field, spectator stands, and trees. We placed four types of obstacles in the central open area, including cubes, rectangular prisms, triangular pyramids, and spheres. The diversity of these geometric shapes provides a testing environment for evaluating the robot’s navigation performance under various obstacle configurations it has not encountered during training.
2. Canyon scenario: This setting replicates a canyon environment, where the central area is densely populated with multiple rock pillar obstacles, forming a concentrated obstacle cluster. The UAV must navigate through this dense obstacle field to reach the other side, presenting a significant challenge to the algorithm’s obstacle avoidance and path planning capabilities.
3. Urban Scenario: To evaluate navigation capabilities in unknown dynamic environments, we constructed an urban scenario featuring seven pedestrians as moving obstacles and three cars of different shapes as static obstacles. Five pedestrians cross the flight path laterally from both sides, while two pedestrian obstacles move along the street, generating interaction scenarios such as crossing, head-on, and side-by-side approaches. All dynamic obstacles move at a constant speed of 0.5m/s. The UAV is restricted to a low-altitude flight zone and cannot fly over obstacles to avoid them.

### 4.2. Results

#### 4.2.1. Static Scenarios

We conducted a comparative evaluation of our method against two other vision-based navigation methods across various scenarios. The first baseline method, SAC+RAE [22], uses a regularized autoencoder (RAE) to encode depth images and then feeds the encoded features, target direction, and UAV velocity into the SAC model to generate control commands. The second baseline method, SAC+CFS [23], introduces a CFS module during the depth-image encoding process to enhance the relevance of the encoded features to the task objective and reduce the influence of non-causal factors. All three methods were trained in a playground scenario until their respective training curves converged.

Table 3 reports the results in the two static scenarios, with the best result in each scene highlighted in bold. Each method was evaluated in 100 episodes. The proposed method achieved the highest success rate and SPL in both scenarios. Compared with SAC+RAE and SAC+CFS, its success-rate gains were 24 and 5 percentage points in the playground and 78 and 15 percentage points in the canyon, respectively.

In both static scenarios, the proposed method achieved the highest success rate and SPL, as well as the shortest additional distance. These results suggest favorable navigation efficiency and cross-scenario transferability across the specific obstacle geometries evaluated in the Playground and Canyon scenarios.

#### 4.2.2. Dynamic Scenario

To evaluate the ability to avoid dynamic obstacles and achieve cross-scene transfer, we further compared our proposed SAC-APF method with SAC-CFS, SAC-RAE, and two additional comparison methods: SAC-CUPRL [49] and CURL-Depth [50]. SAC-CUPRL is the main method proposed in the Depth-CUPRL study, whereas CURL-Depth is the depth-image adaptation of CURL used as a comparison benchmark in that study [49]. All methods were trained in environments with simple structures and short-distance navigation, and were directly transferred to longer, unseen dynamic city maps without the need for retraining, environmental adaptation, or parameter tuning specific to each method.

As shown in Table 4, the success rate of SAC-APF reached 94%, which is 49, 74, 58, and 51 percentage points higher than the success rates of SAC-CFS, SAC-RAE, SAC-CUPRL, and CURL-Depth, respectively. This behavior is consistent with the intended role of the hierarchical structure: the goal-oriented branch controls movement in obstacle-free areas, while the SAC branch is activated when dynamic obstacles enter the forward field of view. Among the evaluated methods, SAC-APF also achieved the highest SPL and the lowest PVR@6 m, suggesting a favorable balance among task completion, path efficiency, and close-range exposure to dynamic obstacles in the tested urban scenario. However, other methods retained advantages in terms of AMOD or JRMS.Since this study employed a unified training and testing protocol, the relatively low success rates of the comparison methods require a reasonable explanation. All methods were trained on short-distance navigation tasks and then directly applied to longer dynamic navigation paths. In this transfer scenario, the baseline methods failed to achieve the performance levels reported in their original papers. No environment-specific adjustment, retraining, or parameter tuning was applied to the comparison methods in the dynamic scenario. The comparison therefore evaluates cross-scenario transfer under consistent experimental conditions.

#### 4.2.3. Ablation Experiment

To investigate the contributions of the APF-based reward mechanism and the CNN-based hierarchical switching mechanism, we evaluated three configurations across the Playground, Canyon, and Urban scenarios. The AE architecture and hyperparameters were kept unchanged across all ablation configurations because the AE served as the common depth-image representation module. SAC+RAE served as the baseline configuration without the proposed APF-based reward mechanism or CNN-based hierarchical switching mechanism. As shown in Table 5, the observed success rates achieved by configurations using APF or CNN were higher than the SAC+RAE baseline results reported in Table 3 and Table 4. The full APF+CNN configuration achieved the highest overall success rate and SPL. For example, in the Playground scenario, the success rate for the configuration using only APF was 0.92, while that for the configuration using only CNN was 0.83. In the Canyon scenario, the corresponding success rates were 0.65 and 0.53, respectively. These descriptive differences indicate that, under the evaluation conditions, the APF-based reward contributes to improved cross-scenario navigation performance.

The JRMS results further show that the APF+CNN configuration obtained the lowest observed values across all three scenarios (Playground: 0.245, Urban: 0.234, and Canyon: 0.392). This pattern suggests that the hierarchical switching mechanism can help reduce velocity oscillations under the evaluated conditions. Configurations without the CNN module produced higher JRMS values in these tests, which is consistent with the intended separation of goal-oriented navigation and local obstacle avoidance.

### 4.3. Real-World Experiment

**Figure 3:**
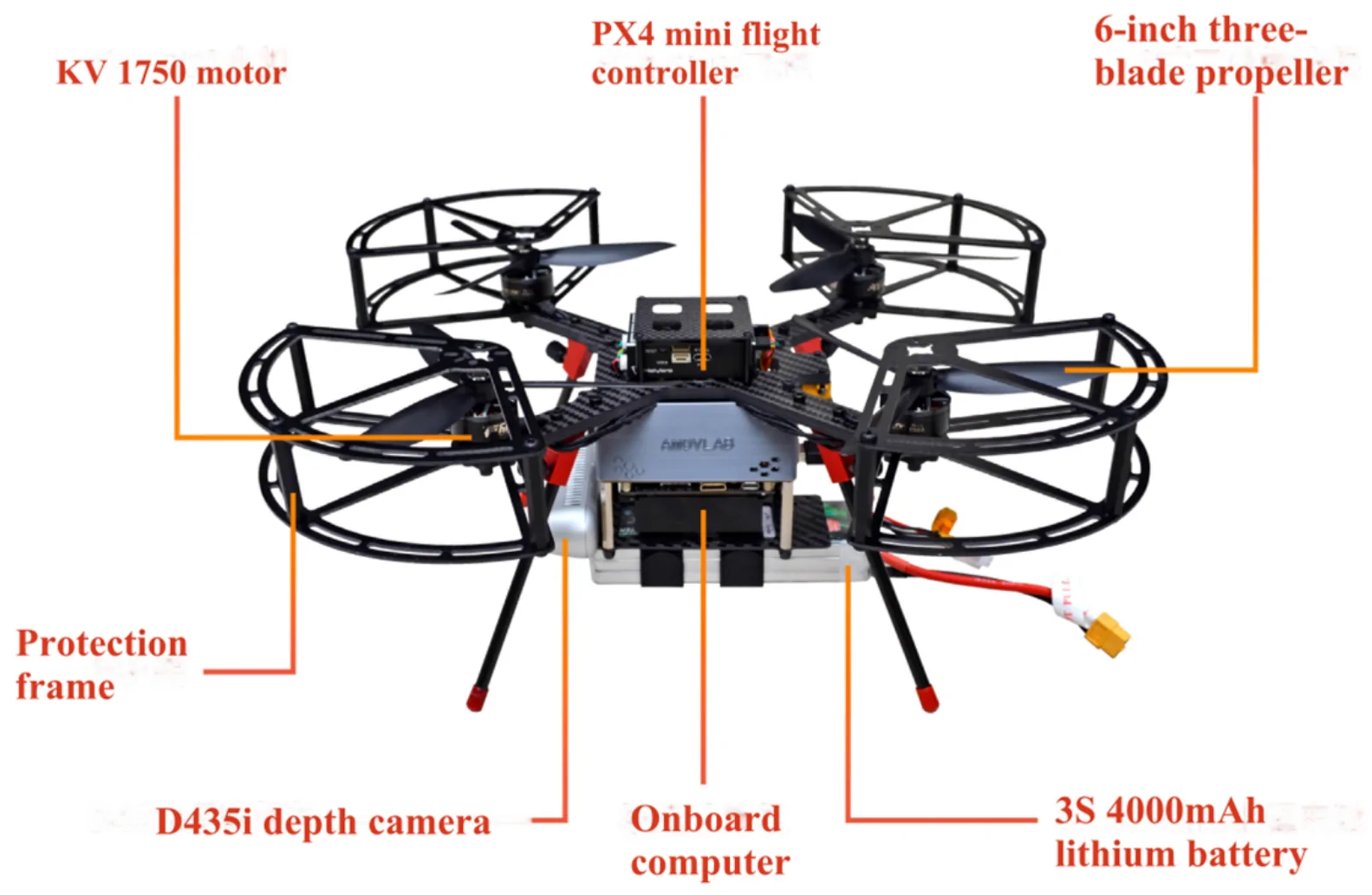
Hardware configuration of the UAV platform used in the real-world experiment.

In the physical experiment, we used the Amov Lab P230 UAV platform as the test vehicle. The UAV was equipped with an Intel RealSense D435i depth camera for local environmental perception and an NVIDIA Jetson Xavier NX onboard computer for real-time state estimation and motion planning. The visual-inertial navigation system (VINS) estimated the UAV’s position, attitude, and velocity using only visual measurements from the D435i camera and inertial measurements from its integrated inertial measurement unit (IMU). No external positioning systems, including motion capture, ultra-wideband positioning, or GPS, were used. The planner did not receive a pre-built map or real-time position information from an external localization system. The hardware configuration is shown in Figure 3.

To verify the feasibility of the proposed method, we constructed a 4m×4m indoor test area containing three cubic obstacles, each approximately 0.8m high and 0.4m wide. A corresponding virtual environment with the same test-area dimensions, number of obstacles, and obstacle layout was constructed in the simulation software.

The start and target positions were defined in the local VINS coordinate frame. The start position was $p_{s}={\left[x_{s},y_{s},z_{s}\right]}^{T}\;m$, and the target position was $p_{g}={\left[x_{g},y_{g},z_{g}\right]}^{T}\;m$. The target position was specified before take-off in the local VINS coordinate frame and was not provided by an external positioning system.

**Figure 4:**
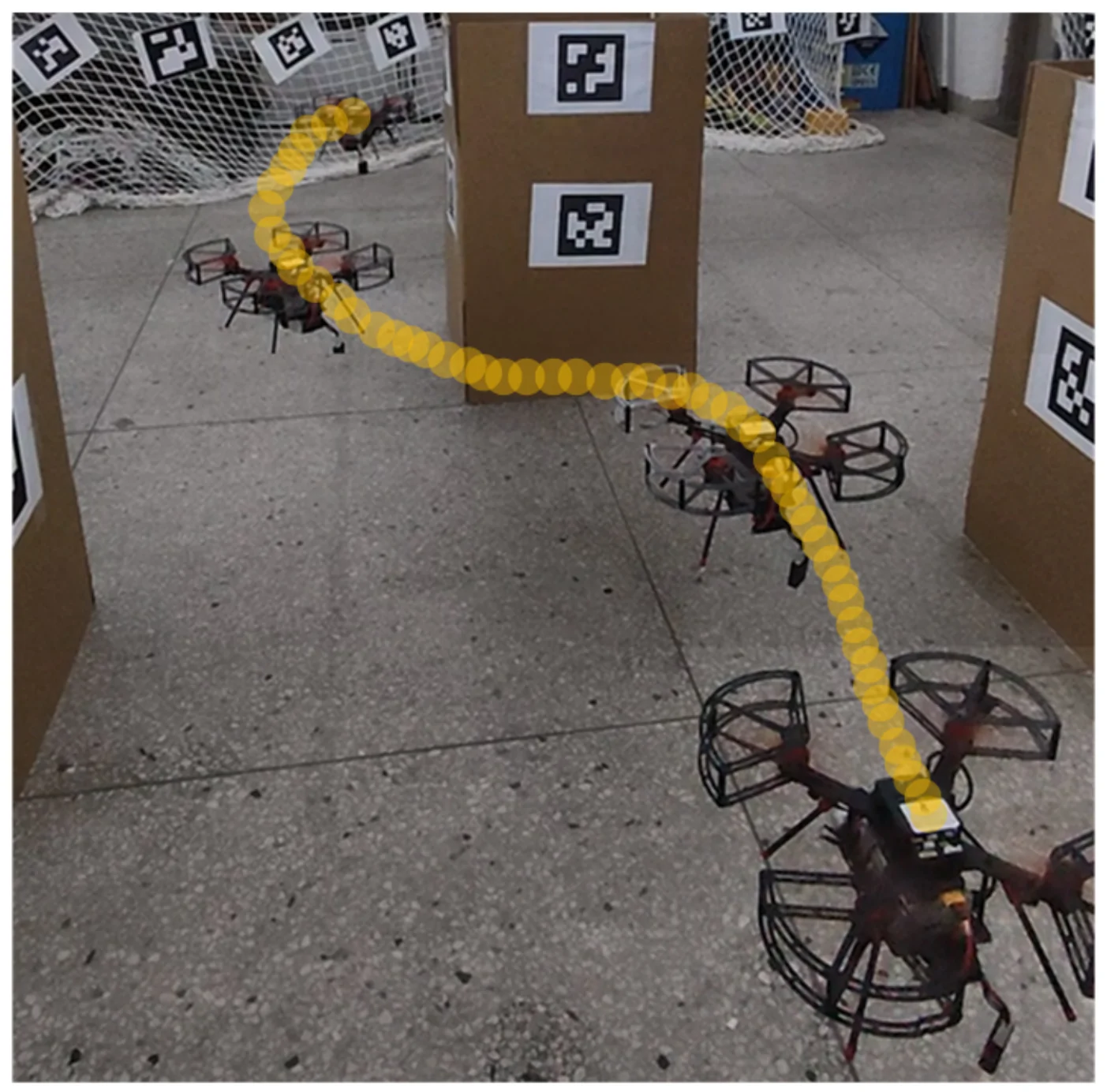
Real-world UAV trajectory recorded from the synchronized onboard VINS state. The yellow line represents the physical UAV flight trajectory mapped into the corresponding virtual environment.

During the physical flight, the UAV state estimated by the onboard VINS was synchronized with the corresponding virtual environment. The simulation software recorded the synchronized position sequence to generate the flight trajectory. Therefore, the trajectory shown in Figure 4 represents the recorded VINS state history of the physical UAV mapped into the corresponding virtual environment; it is not an independently generated simulation trajectory or an externally measured ground-truth trajectory. The control commands applied to the physical UAV had corresponding representations in the virtual environment, enabling synchronized visualization and trajectory recording.

The artificial markers visible in the experimental images were used only as visual references for displaying the experimental scene. They were not used for UAV localization, navigation decisions, VINS initialization, scale estimation, or trajectory evaluation.

As shown in Figure 4, the UAV moved directly toward the target when it was far from the obstacles and switched to the obstacle-avoidance commands generated by the policy when approaching the obstacles. The UAV successfully avoided the three obstacles and reached the target located behind the final obstacle. This experiment is regarded as a preliminary feasibility demonstration of onboard deployment rather than comprehensive evidence of robustness in large-scale real-world environments. The simulation and real-world experiments are demonstrated in Video S1.

## 5. Discussion

Although the proposed method achieved favorable results under the evaluated conditions, several limitations remain. First, the current experimental evaluation is limited to single-drone navigation tasks. In practical applications, collaborative navigation involving multiple drones presents additional challenges, including collision avoidance between drones, distributed decision-making, communication constraints, and task coordination and allocation. Second, the current UAV platform is equipped with only one forward-facing depth camera, resulting in a limited field of view that affects its ability to perceive obstacles and perform omnidirectional maneuvers. Third, the experiments were conducted in a relatively controlled indoor environment; the robustness of this method under more complex conditions—such as varying lighting, sensor noise, and fast-moving obstacles—requires further investigation. Fourth, the current training and evaluation scheme is primarily designed to assess the feasibility of the proposed architecture in the test environment. Therefore, the reported results should be regarded as a preliminary performance evaluation rather than a comprehensive validation. Future research will adopt a more comprehensive training and evaluation scheme to further evaluate the performance consistency and applicability of the proposed method under a wider range of conditions. Future work will also extend this framework to support multi-drone collaboration.

## 6. Conclusions

This study proposes a vision-based hierarchical navigation method for unmanned aerial vehicles (UAVs) operating in unstructured environments. The proposed framework combines an APF-based reward function, a CNN-based switching mechanism, and deep reinforcement learning (DRL) to enhance obstacle avoidance, navigation efficiency, and policy smoothness. In the static Playground and Canyon scenarios, the proposed method achieved the highest success rate and SPL and the shortest additional distance among the evaluated methods. Under the common transfer protocol from short-range training to an unseen long-range dynamic Urban scenario, it achieved a 94% success rate, the highest SPL, and the lowest PVR@6 m, while other methods retained advantages in individual clearance or smoothness metrics. These results indicate that the proposed control architecture provides a favorable balance among completion reliability, path efficiency, and dynamic-obstacle exposure under the evaluated conditions. The preliminary indoor flight experiment indicates the feasibility of deploying the proposed method onboard a physical UAV equipped with visual and inertial sensors under the evaluated conditions. The current evaluation remains limited to a single UAV with a forward-facing depth camera. Future work will investigate multi-UAV coordination and multi-directional perception in larger and more dynamic environments. Furthermore, the proposed technical framework is not limited to drones but can be extended to other omnidirectional mobile platforms, such as underwater robots and Mecanum-wheeled robots, although platform-specific modeling and experimental validation will still be required in specific operational environments. 

## Figures and Tables

**Table 1:** Hyperparameters for CNN training.

Parameter Name	Value
Optimizer	Adam
Loss Function	Binary Cross-Entropy
Learning Rate	0.001
Batch Size	64
Epochs	500
Total Samples	40,000

**Table 2:** Hyperparameters for policy training.

Parameter Name	Value
Depth Observation Size	4×84×84
Latent Feature Dimension	50
Action Dimension	3
Actor Hidden Layers	[1024,1024]
Critic Hidden Layers	[1024,1024] (Twin Q-networks)
Replay Buffer Size	20,000
Batch Size	256
Discount Factor	0.99
Optimizer	Adam
Actor Learning Rate	$10^{-4}$
Critic Learning Rate	$10^{-4}$
Encoder/Decoder Learning Rate	$10^{-4}$
Alpha Learning Rate	$10^{-5}$
Target Entropy	−3
AE Reconstruction Loss	Mean Squared Error
Network Update Frequencies (Critic/Encoder/Decoder)	1/1/1
Target Update Coefficient (τ)	0.005
Actor Log Stddev Bounds	[−10,2]
Total Episodes	4000
Warmup Episodes	200
Max Timesteps	200

**Table 3:** Performance comparison in static scenarios.

Scene	Method	Success Rate	SPL	Extra Distance (m)	Average Speed (m/s)
Playground	SAC+RAE	0.75	0.742	2.381/1.050	1.379/0.049
	SAC+CFS	0.94	0.935	1.367/1.775	1.433/0.035
	Our method	**0.99**	**0.990**	**0.803/0.361**	**1.490/0.004**
Canyon	SAC+RAE	0.06	0.032	73.518/6.519	1.331/0.711
	SAC+CFS	0.69	0.654	6.958/7.912	**1.527/0.062**
	Our method	**0.84**	**0.833**	**2.435/2.715**	1.478/0.006

*Note:* Slash-separated values are reported as mean/standard deviation over the evaluation episodes. Bold values indicate the best result for each metric within each scene.

**Table 4:** Performance comparison in dynamic scenarios.

Method	Success Rate	SPL	Extra Dist. (m)	Avg. Speed (m/s)	JRMS	AMOD (m)	PVR@6 m
SAC-APF (ours)	**94%**	**0.8875**	2.9416	1.9405	0.1948	4.1030	**2.04% ± 3.90%**
SAC-CFS	45%	0.4499	3.6957	1.9400	**0.0755**	4.5436	6.33% ± 5.78%
SAC-RAE	20%	0.2000	**2.2994**	**2.0561**	0.5673	3.2316	11.98% ± 7.00%
SAC-CUPRL	36%	0.3522	8.6922	1.6324	0.5354	**4.8804**	6.23% ± 3.10%
CURL-Depth	43%	0.4287	5.9659	1.6455	0.7531	4.3672	4.35% ± 2.44%

*Note:* Bold values indicate the best result for each metric.

**Table 5:** Ablation study results.

Scene	APF	CNN	Success Rate	SPL	Average Speed	JRMS
Playground	✓	–	0.92	0.847	1.441/0.014	0.506/0.028
	–	✓	0.83	0.810	**1.499/0.041**	0.372/0.100
	✓	✓	**0.99**	**0.990**	1.490/0.004	**0.245/0.037**
Canyon	✓	–	0.65	0.544	1.435/0.013	0.626/0.017
	–	✓	0.53	0.530	**1.488/0.020**	0.759/0.075
	✓	✓	**0.84**	**0.833**	1.478/0.006	**0.392/0.049**
Urban	✓	–	0.95	0.934	1.450/0.013	0.584/0.017
	–	✓	0.45	0.447	**1.485/0.016**	0.515/0.060
	✓	✓	**0.97**	**0.963**	1.460/0.005	**0.234/0.089**

*Note:* Slash-separated values are reported as mean/standard deviation over the evaluation episodes. Bold values indicate the best result for each metric within each scene.

## Data Availability

The code is available in an online repository (https://github.com/kszkad/UAV-NAVIGATION-VIA-DRL, accessed on 13 August 2026).

## Supplementary Materials

The following supporting information can be downloaded at: https://www.mdpi.com/article/10.3390/s26165196/s1, Video S1: UAV navigation simulation and real-world experiments.

## Abbreviations

The following abbreviations are used in this manuscript:

UAV	Unmanned aerial vehicle
DRL	Deep reinforcement learning
CNN	Convolutional neural network
APF	Artificial potential field
SAC	Soft Actor–Critic
AE	Autoencoder
POMDP	Partially observable Markov decision process
SPL	Success weighted by path length
JRMS	Root mean square jerk
AMOD	Average minimum obstacle distance
PVR	Proximity violation rate
VINS	Visual–inertial navigation system
